# Effect of Ca_v_β Subunits on Structural Organization of Ca_v_1.2 Calcium Channels

**DOI:** 10.1371/journal.pone.0005587

**Published:** 2009-05-18

**Authors:** Evgeny Kobrinsky, Parwiz Abrahimi, Son Q. Duong, Sam Thomas, Jo Beth Harry, Chirag Patel, Qi Zong Lao, Nikolai M. Soldatov

**Affiliations:** National Institute on Aging, National Institutes of Health, Baltimore, Maryland, United States of America; University of Cincinnati, United States of America

## Abstract

**Background:**

Voltage-gated Ca_v_1.2 calcium channels play a crucial role in Ca^2+^ signaling. The pore-forming α_1C_ subunit is regulated by accessory Ca_v_β subunits, cytoplasmic proteins of various size encoded by four different genes (Ca_v_β_1_ - β_4_) and expressed in a tissue-specific manner.

**Methods and Results:**

Here we investigated the effect of three major Ca_v_β types, β_1b_, β_2d_ and β_3_, on the structure of Ca_v_1.2 in the plasma membrane of live cells. Total internal reflection fluorescence microscopy showed that the tendency of Ca_v_1.2 to form clusters depends on the type of the Ca_v_β subunit present. The highest density of Ca_v_1.2 clusters in the plasma membrane and the smallest cluster size were observed with neuronal/cardiac β_1b_ present. Ca_v_1.2 channels containing β_3_, the predominant Ca_v_β subunit of vascular smooth muscle cells, were organized in a significantly smaller number of larger clusters. The inter- and intramolecular distances between α_1C_ and Ca_v_β in the plasma membrane of live cells were measured by three-color FRET microscopy. The results confirm that the proximity of Ca_v_1.2 channels in the plasma membrane depends on the Ca_v_β type. The presence of different Ca_v_β subunits does not result in significant differences in the intramolecular distance between the termini of α_1C_, but significantly affects the distance between the termini of neighbor α_1C_ subunits, which varies from 67 Å with β_1b_ to 79 Å with β_3_.

**Conclusions:**

Thus, our results show that the structural organization of Ca_v_1.2 channels in the plasma membrane depends on the type of Ca_v_β subunits present.

## Introduction

Voltage-gated Ca_v_1.2 calcium channels react to membrane depolarization by creating a rapid and transient increase in intracellular free Ca^2+^ concentration, thereby playing an essential role in initiation of calcium signaling in a wide variety of cells [1]. In order to exhibit this function, Ca_v_1.2 calcium channels require association of the pore-forming α_1C_ subunit with accessory Ca_v_β and α_2_δ subunits as well as calmodulin. Calcium channels are clustered rather than evenly distributed along the surface membrane of neurons [2]–[4] and cardiac myocytes [5]–[7]. Single-molecule imaging of the functional recombinant EYFP_N_-α_1C_/β_2a_/α_2_δ channels revealed clusters composed of ∼40 channels [8]. In neuronal cell bodies and proximal dendrites in hippocampus and cerebral cortex, Ca_v_1.2 clusters of 1.5–2 µm in diameter were observed with anti-α_1C_ antibody [9]. Using electron microscopy in bird and amphibian cardiac muscle [5], [6] and immuno-gold labeling in mammalian ventricular myocytes [7], [10] it was shown that Ca_v_1.2 clusters are loosely tethered to ryanodine receptors (RyR) of the sarcoplasmic reticulum. Although association of calcium channels and ryanodine receptors appears to be weaker in cardiac myocytes than in skeletal muscle [11] and may involve different mechanisms of coupling [12], Ca_v_1.2 clustering is essential for excitation-contraction coupling [13], [14].

Little is known about the factors affecting the structure of Ca_v_1.2 clusters or the mechanisms of their formation. Because the carboxyl-terminal “IQ” region of α_1C_ mediates the calmodulin-dependent Ca^2+^-induced inactivation of the channel [15]–[18], it is reasonable to suggest that both calmodulin and the cytoplasmic 750-amino acid C-tail of α_1C_ have a role in the formation and maintenance of the Ca_v_1.2 clusters. Indeed, a splice variant of α_1C_ (α_1C,86_) deprived of IQ does not show a distinct tendency to form clusters [19]. The role of IQ sequences in intermolecular interactions between neighboring α_1C_ molecules was experimentally confirmed in recent diffraction study [20]. The impact of bulky cytoplasmic Ca_v_β subunits on Ca_v_1.2 structure and clustering is not known. Ca_v_β subunits are important differential modulators of the electrophysiological properties of calcium channels [21]–[23]. These peripheral proteins of variable size are encoded by four different genes (Ca_v_β_1_ - β_4_), some of them being subject to alternative splicing [24]. They have a common binding site in the cytoplasmic linker between repeats I and II of α_1C_ known as the α-interaction domain (AID) [25]. Here, we applied total internal fluorescence reflection (TIRF) and three-color FRET microscopy to assess the effects of Ca_v_β on cluster size and density of Ca_v_1.2 as well as to measure inter- and intramolecular distances between the N- and C-termini of α_1C_ and the N-tails of β_1b_, β_2d_ and β_3_. Our results demonstrated that Ca_v_1.2 channels form plasma membrane clusters and revealed the effect of the type of Ca_v_β present on molecular distances and packing of the channels.

## Results

### Differential effect of Ca_v_β subunits on cluster organization of Ca_v_1.2 channels

Ca_v_1.2 calcium channels retain functional activity after fusion of fluorescent proteins to the N- and C-termini of α_1C_ and to the N-terminus of Ca_v_β. In our experiments, we labeled α_1C_ at the N-tail with monomeric mVenus (Vα_1C_) and/or at the C-tail with monomeric mCerulean (α_1C_ C) [26]. To investigate the effect of Ca_v_β subtype on size and density of Ca_v_1.2 clusters, we chose three major Ca_v_β variants, neuronal/cardiac β_1b_ [27], cardiac β_2d_ [28], [29] and neuronal/cardiac/vascular β_3_ [30]–[32], which is the predominant Ca_v_β subunit in vascular smooth muscle cells [31], [33]. The more commonly used β_2a_ was excluded from the experiments because its N-tail is palmytoylated and anchored to the inner leaflet of the plasma membrane.

**Figure 1:**
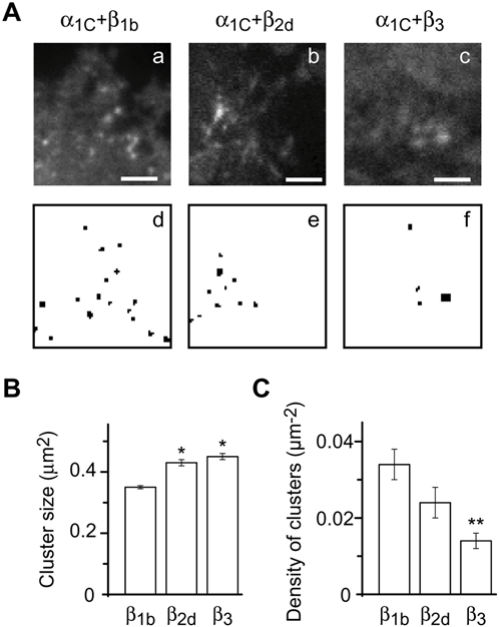
Effect of Ca_v_β subunits on cluster organization of Ca_v_1.2 channels. (*A*) TIRF images (*a–c*) and wavelet-derived clusters (*d–f*) of Ca_v_1.2 channels containing β_1b_ (*a*,*d*), β_2d_ (*b*,*e*) or β_3_ (*c*,*f*). Scale, 4.5 µm. (*B*) Dependence of the average size of Ca_v_1.2 clusters on the type of Ca_v_β present. β_1b_, mean size±SEM, 0.360±0.005 µm^2^ (number of clusters analyzed m = 1253); β_2d_, 0.430±0.013 mm^2^ (m = 270); β_3_, 0.450±0.017 µm^2^ (m = 205). *, *P*<0.001 relative to β_1b_. (*C*) Dependence of the number of Ca_v_1.2 clusters (normalized to the area measured and defined as density) on the type of Ca_v_β present. β_1b_, mean number±SEM, 0.034±0.004 mm^−2^ (number of cells n = 27); β_2d_, 0.024±0.04 µm^−2^ (n = 30); β_3_, 0.014±0.02 µm^−2^ (n = 22). **, *P*<0.01 relative to β_1b_. Vα_1C_ was co-expressed with α_2_δ and indicated Ca_v_β in COS1 cells.

Fluorescent microscopy is a convenient approach to detect clusters of recombinant calcium channels as fluorescent foci or groupings of labeled molecules [34]. In this study, we used TIRF microscopy to visualize Ca_v_1.2 clusters on the basal plasma membrane. Wavelet transform was used for the detection of clusters (see Methods and Figure 1*A*) to estimate the effect of the type of Ca_v_β present on the Ca_v_1.2 clusters size (Figure 1*B*) and density (defined here as number of clusters per µm^2^ of the plasma membrane, Figure 1*C*). The smallest Ca_v_1.2 clusters were observed with β_1b_ present. Ca_v_1.2 clusters were significantly (*P*<0.001) larger with β_2d_ (by ∼20%) and β_3_ present (by ∼30%) (Figure 1*B*). We also found that the average density of the Vα_1C_/β_1b_ clusters in the plasma membrane was 2.5 times higher (*P*<0.01) than Vα_1C_/β_3_, with β_2d_ again taking an intermediate value (Figure 1*C*). Thus, Ca_v_β subunits differentially regulate the architecture of the Ca_v_1.2 clusters.

**Figure 2:**
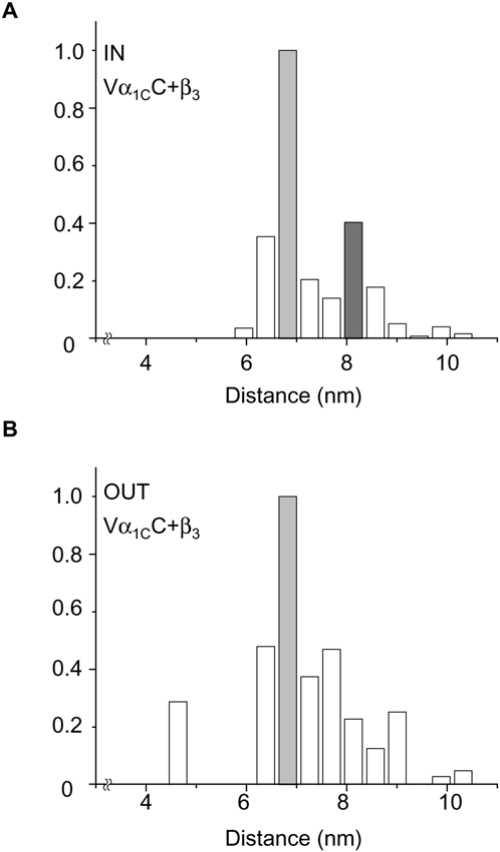
Intramolecular vs. intermolecular FRET in Vα_1C_C revealed in TIRF images. Vα_1C_C, α_2_δ and β_3_ were co-expressed in COS1 cells. Two-color FRET was measured in TIRF images and converted into distances *r* between V and C as described in Methods. Shown are normalized cumulative histograms (n = 11) for *r* calculated for ROI inside clusters (*A*, total number of pixels m = 231) and outside clusters (*B*, m = 3908) identified by wavelet transform. The same intramolecular (*r*_V-C_) distance ≈6.8 nm (light gray bars) was observed both inside and outside clusters, while intermolecular (*r*_V∼C_) distance ≈8.1 nm was observed only in clusters (dark gray).

In principle, the close proximity of channels within a cluster may generate intermolecular FRET between the V and C fluorophores of neighboring Vα_1C_C channels. This intermolecular FRET should be absent outside of clusters, where only intramolecular FRET should occur. The Vα_1C_C/α_2_δ/β_3_ channel was expressed in COS1 cells and two-color TIRF-FRET was measured inside and outside of clusters identified by wavelet transform. Based on FRET efficiency, a V-C distance (*r*)-frequency histogram of the total number of pixels within clusters revealed a possible bi-modal distribution, where a second (intermolecular) component of FRET is seen within clusters (Figure 2*A*) but not outside of the clusters (Figure 2*B*). Because TIRF microscopy captures only a small fraction of the cell plasma membrane, we used epifluorescent three-color FRET microscopy to quantitatively analyze the effect of Ca_v_β subtype on inter- and intra-molecular distance of Ca_v_1.2 channels.

### The type of Ca_v_β present does not affect intramolecular distance between the N- and C-termini of the α_1C_ subunit

**Figure 3:**
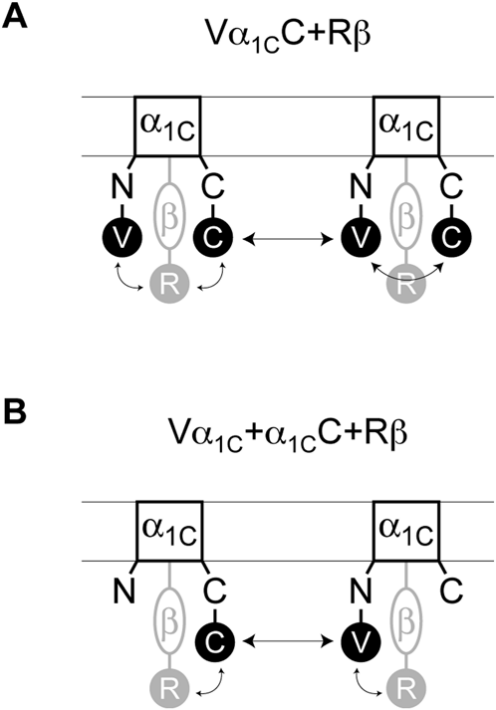
Investigated combinations of the labeled α_1C_ and β subunits for three color FRET measurements. Vα_1C_C and Rβ (A) and Vα_1C_, α_1C_C and Rβ (B) were co-expressed with α_2_δ (not shown). Arrows indicate revealed intramolecular and intermolecular distances.

**Figure 4:**
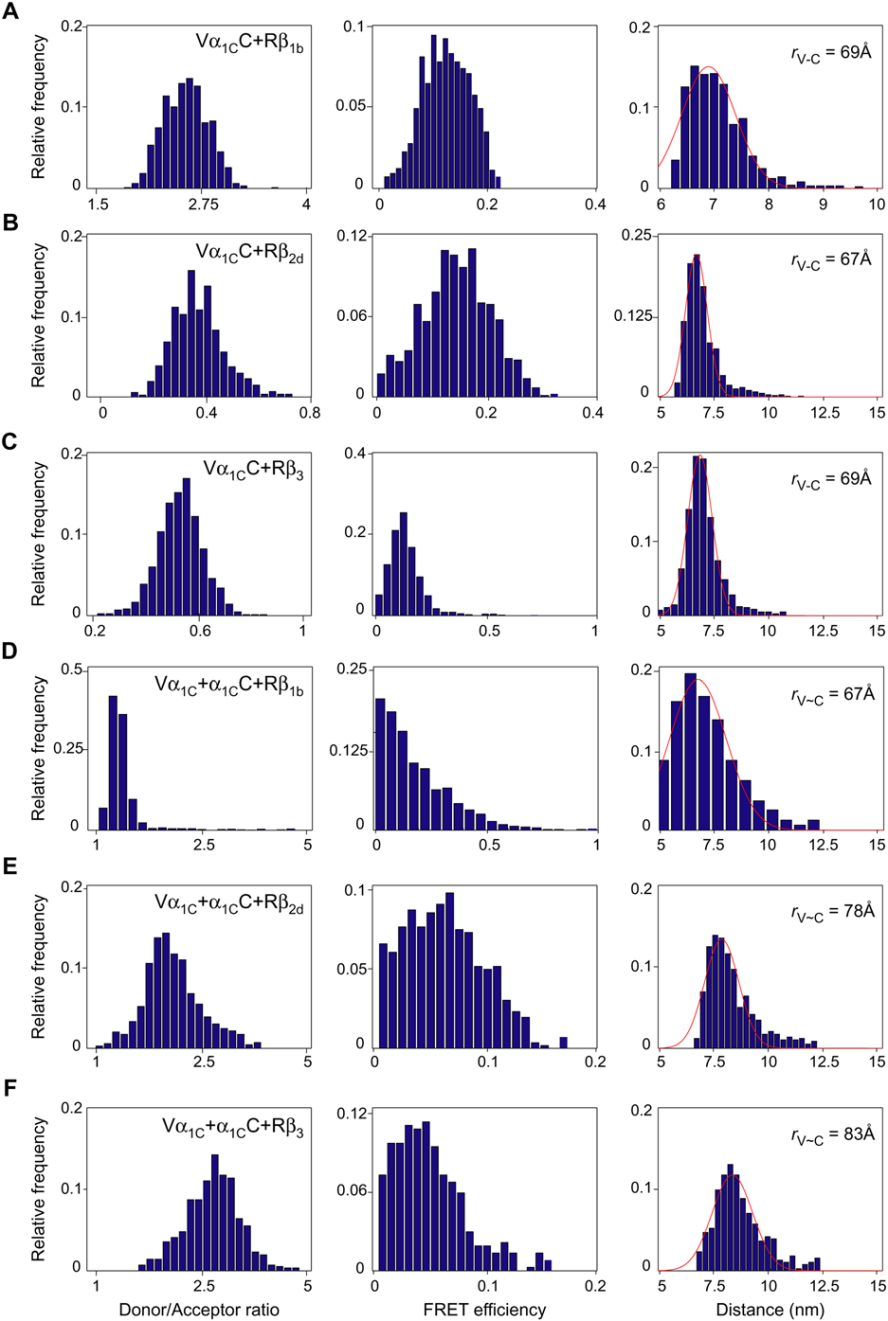
Estimation of distance *r* between fluorophores fused to the N- and/or C-termini of the α_1C_ subunit. (*A–C*) Intramolecular FRET recorded with Vα_1C_C. (*D–F*) Intermolecular FRET recorded with Vα_1C_+α_1C_C. Channels were co-expressed in COS1 cells with α_2_δ and Rβ_1b_ (*A* and *D*), Rβ_2d_ (*B* and *E*) or Rβ_3_ (*C* and *F*). Shown are representative of histograms calculated from single exemplary cells for donor/acceptor ratio (left column), FRET efficiency (middle column) and distance (right column). Relative frequency was calculated for total number of pixels in ROI as described in Methods. The red solid line is the best fit to a Gaussian distribution with indicated means for *r*_V-C_ and *r*_V∼C_.

We investigated the effect of Ca_v_β subtype on molecular distances in Ca_v_1.2 channels by three-color FRET between Vα_1C_C and tagRFP (Rβ) fused to N-termini of β_1b_, β_2d_ and β_3_. The advantage of three-color FRET cell microscopy applied to multisubunit complexes is that the method simultaneously detects the relative arrangement of the three different fluorophores (C, V and R) at a distance ≤2×*R*_o_, where *R*_o_ is the Förster radius (*R*_o(C-V)_ = 53 Å; *R*_o(V-R)_ = 58 Å; *R*_o(C-R)_ = 51 Å). Both mCerulean and mVenus are close analogs of GFP and can be approximated by a cylinder of 32×48 Å [35]. However, tagRFP [36], a monomeric analog of eqFP611, is larger in size and can be approximated by a cylinder of 34×54 Å [37]. Use of monomeric forms of fluorescent proteins excludes artifacts due to dimerization after expression [38]. The labeled constructs were co-expressed with α_2_δ in two different combinations as shown in Figure 3, and three-color FRET was measured using a multicube system [39]. Although membrane potential was not controlled during experiments, it was found to be on average −10.0±3.3 mV (n = 5) indicating that channels were predominantly in an inactivated state. In each fluorescent cell image, region of interest (ROI) was determined using a standard procedure as described earlier [40]. Within this ROI, only pixels with donor/acceptor ratio from 0.2 to 5 (Figure 4, left panels) were selected for further analysis [41]. FRET efficiency was determined according to [42] (Figure 4, middle panels) and then converted to the distance (*r*) between donor and acceptor (right panel) according to [43].

Results of our measurements revealed that the tested Ca_v_β subunits did not affect intramolecular distance between the N- and C-termini of α_1C_. Measurement of FRET in the double-labeled Vα_1C_C co-expressed with α_2_δ and Rβ_1b_, Rβ_2d_ or Rβ_3_ showed that the intramolecular distance *r*_C-V_ between V and C did not vary significantly and was on average 68–69 Å, independent of the type of co-expressed Ca_v_β (Figure 4, *A–C*; see Table 1 for statistics).

**Table 1:** Intra- and intermolecular distances between the Ca_v_1.2 calcium channel α_1C_ and β subunits measured by three-color FRET microscopy.

Channel subunits	Measured distances (*r*)	β_1b_	β_2d_		β_3_	
		*r*, Å	*r*, Å	*c*	*r*, Å	*c*
Vα_1C_C+Rβ	*r*_C-V_	68±1 (17)	68±2 (13)	0.90±0.37	69±1 (19)	0.60±0.05
	*r*_C∼V_		72±3 (5)	1.27±0.54	77±3 (6)	1.16±0.17
	*r*_V-R_	95±3 (13)	99±3§ (8)	1.70±0.27	90±2 (19)	0.97±0.20
	*r*_V∼R_		107±1 (3)	2.52±0.17	100±2 (15)	1.72±0.23
	*r*_C-R_	85±2‡ (13)	84±2 (13)		79±1 (14)	0.70±0.10
	*r*_C∼R_				85±1 (10)	1.55±0.07
Vα_1C_+α_1C_C+Rβ	*r*_C∼V_	67±1* (26)	72±2 (13)		79±4 (10)	
	*r*_V-R_	90±2 (26)	90±2 (13)		90±5 (10)	
	*r*_C-R_	78±1† (26)	86±2 (6)		80±4 (8)	

* *P*<0.002 vs. β_3_.

† *P*<0.05 vs. β_2d_.

‡ *P*<0.05 vs. β_3_.

§ *P*<0.05 vs. *r*_V-R_ in Vα_1C_+α_1C_C+Rβ_2d_.

FRET efficiency between the indicated fluorophores fused to the α_1C_ and β_1b_, β_2d_ or β_3_ subunits was measured in the plasma membrane of expressing COS1 cells and fitted to a Gaussian function. In cases when the routine curve fit showed two significantly different Gaussian distributions, the corresponding dispersion coefficients *c* (mean±SEM) are shown for both distances (see Experimental Procedures). V – mVenus, C- mCerulean, R – tagRFP. Shown values of *r* are mean±SEM. Number of cells is shown in parentheses.

Estimation of *r*_C-V_ in the absence of Ca_v_β was not possible because of poor plasma membrane targeting by Vα_1C_C/α_2_δ under such conditions. To overcome this problem, we co-expressed Vα_1C_C and α_2_δ with tagRFP-labeled β_2_CED, a 42-amino acid fragment of β_2_ subunits which does not bind to AID, but interacts with the IQ region of the α_1C_ subunit C-terminus, facilitates voltage gating and stimulates surface expression of the channel [44]. Results of FRET measurements showed *r*_C-V_ = 68±1 Å (n = 22), essentially the same distance as that estimated when AID was occupied by Ca_v_β. Taken together, these results of our study suggest that type of Ca_v_β subunits present does not significantly affect the intramolecular distance between the N- and C-termini of α_1C_ in Ca_v_1.2 calcium channels.

### Intermolecular distance between the α_1C_ subunit N- and C-termini depends on the type of Ca_v_β present

**Figure 5:**
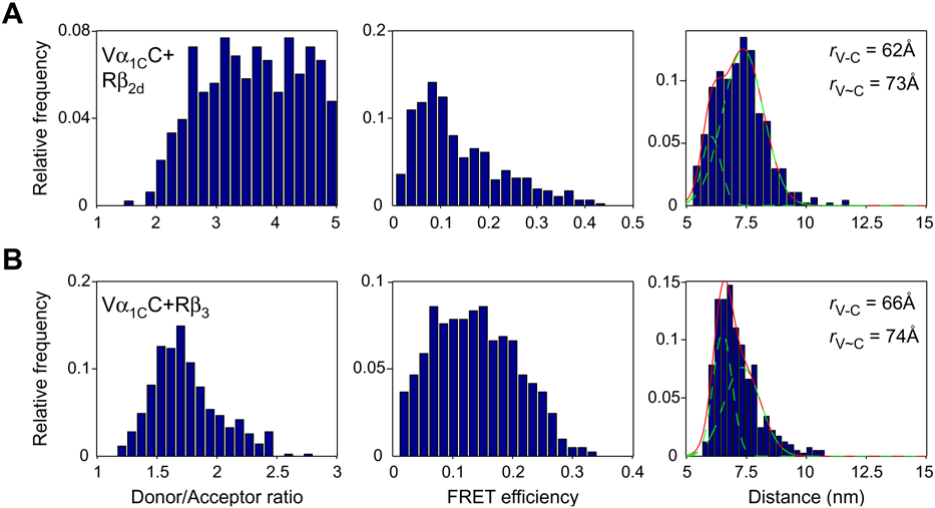
Intramolecular vs. intermolecular FRET in Vα_1C_C. The Vα_1C_C subunit was co-expressed in COS1 cells with α_2_δ and Rβ_2d_ (*A*) or Rβ_3_ (*B*). Shown are histograms of donor/acceptor ratio (left column), FRET efficiency (middle column) and distance (right column) determined in the plasma membrane region of two representative COS1 cells. The red solid line is the best fit to a sum of two Gaussian distributions with indicated means (green dotted lines) for intramolecular (*r*_C-V_) and intermolecular FRET (*r*_C∼V_).

Fitting of FRET data obtained with β_2d_ and β_3_ to a sum of two Gaussian distributions (Table 1) revealed a statistically significant second component of Vα_1C_C FRET (Figure 5). Arising from neighboring Vα_1C_C molecules, this FRET provided estimates for the intermolecular distances (*r*_C∼V_) that were significantly different for β_2d_ (72±3 Å, n = 5) and β_3_ (77±3 Å, n = 6). To verify our intermolecular distance measurements, we co-expressed a mixture of Vα_1C_ and α_1C_C along with Rβ_1b_, Rβ_2d_ or Rβ_3_ (Figure 4, *D–F*). Any FRET between V and C in this recombinant system must be intermolecular FRET between termini of neighboring channels. Results, presented in Table 1, showed that intermolecular distances *r*_C∼V_ measured in these complexes with β_2d_ (72±2 Å, n = 13) and β_3_ (79±4 Å, n = 10) are not significantly different from the *r*_C∼V_ values measured under the same conditions with Vα_1C_C. With β_1b_, the intermolecular distance *r*_C∼V_ measured between Vα_1C_ and α_1C_C was 67±1 Å (n = 26), a value not significantly different from the estimate for intramolecular Vα_1C_C distance (*r*_V-C_ = 68±1 Å, n = 17). This explains why the data obtained in the presence of β_1b_ were best fitted by a single Gaussian distribution. Thus, unlike β_2d_ and β_3_, in the presence of β_1b_ the inter- and intramolecular distances appear to be similar.

**Figure 6:**
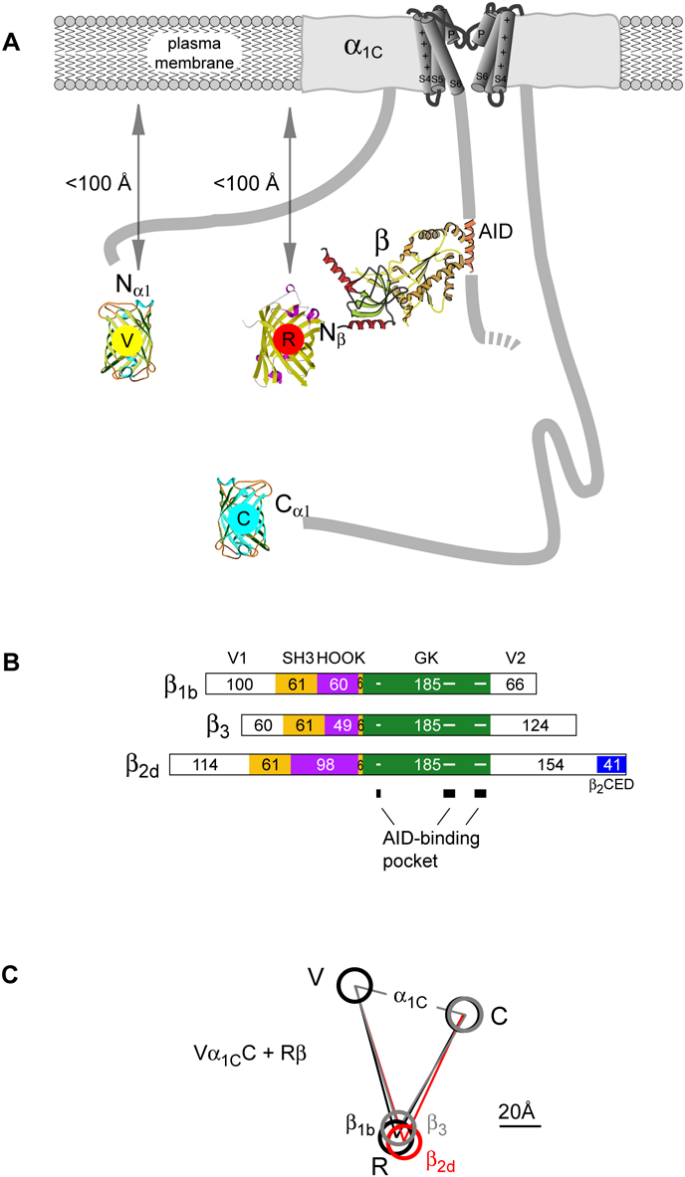
Molecular distances between the N- and C-termini of α_1C_ and the Ca_v_β-subunit N-tail of β_1b_, β_2d_ and β_3_. (*A*) Schematic representation of Vα_1C_C with Rβ arranged under a vertically sliced α_1C_. The structures of TagRFP and Ca_v_β core MAGUK region were drawn based on PDB codes 1uisA [37] and 1t0j [62], respectively. FRET measurements with ECFP-labeled plekstrin homology domain in the inner leaflet of the plasma membrane [40], [63] showed that the N terminal tags of both the α_1C_ and Ca_v_β subunits are located within the 2× Förster distance (<100 Å for ECFP/EYFP) from the plasma membrane. (*B*) Schematic representation of the domain organization of β_1b_, β_2d_ and β_3_ aligned in regard to AID-binding guanylate kinase (GK) domain (green). Yellow box indicates the Src homology 3 (SH3) domain, purple the variable HOOK region, and blue the β_2_CED [44]. Number of amino acids is shown inside boxes. Amino acids involved in AID-binding pocket are marked in GK by three horizontal lines (for details see [62], [64], [65]). (*C*) Schematic representation of the results of simultaneous measurements of the molecular distances between three fluorophores shown in panel (*A*) in Vα_1C_C/α_2_δ/Rβ in the presence of Rβ_1b_ (black lines), Rβ_3_ (gray lines) and Rβ_2d_ (red lines).

The measurements with a mixture of Vα_1C_ and α_1C_C confirm that Ca_v_1.2 calcium channels containing β_1b_, β_2d_ or β_3_ subunits are in close proximity to each other, thus supporting their clustering in the plasma membrane. The distance *r*_C∼V_ between the N- and C-termini of the neighbor α_1C_ subunits depends on the type of Ca_v_β. In the presence of β_1b_, the distance *r*_C∼V_ (67±1 Å) is 1.2 nm smaller (*P*<0.002) than with β_3_ (79±4 Å), while *r*_C∼V_ estimated in the presence of β_2d_ (72±2 Å) is of an intermediate value. Subsequent measurements of three-color FRET between Rβ and the fluorophores of the α_1C_ subunit added more certainty to this general picture (Figure 6*A*).

### FRET between tagRFP-labeled Ca_v_β and mCerulean/mVenus-labeled α_1C_

The three Ca_v_β subunits selected for our study vary in molecular mass (β_1b_, 53.2 kDa; β_2d_, 73.5 kDa; β_3_, 54.5 kDa) and in the size of the variable N-terminal (V1), central (HOOK) and C-terminal (V2) regions (see Figure 6*B*). There are large differences between the three Ca_v_β subunits in variable regions on both sides of the AID-binding pocket, which anchors Ca_v_β to the I–II linker of α_1C_ (Figure 6*A*). In spite of that, the intramolecular distance *r*_V-R_ between Rβ and Vα_1C_ estimated in all tested three-color FRET combinations, including single- or double-labeled α_1C_ (Vα_1C_+α_1C_C+Rβ, Vα_1C_C+Rβ), was not significantly different for all tested Ca_v_β subunits except for Rβ_2d_ (see § in Table 1) Although the average distances *r*_C-R_ between Rβ and Vα_1C_C were significantly different for Rβ_1b_ (85±2 Å, n = 13) and Rβ_3_ (79±1 Å, n = 14), they were not significantly different between Rβ and α_1C_C. A superposition of all three simultaneously measured arrangements between Rβ and Vα_1C_C (Figure 6*C*) illustrates differences in the positions of Rβ subunits as reflected by statistically significant differences in *r*_V-R_ and *r*_C-R_ (Table 1).

Fitting to a sum of two Gaussian distributions did not reveal the second (intermolecular) component of FRET between Vα_1C_C and Rβ_1b_ (Table 1). However in the case of Rβ_3_ two intermolecular FRET components were clearly observed, one corresponding to the distance *r*_V∼R_ = 100±2 Å (in 15 of 19 cells) and the other corresponding to *r*_C∼R_ = 85±1 Å (in 10 of 14 cells). In the presence of Rβ_2d_, the latter component was not observed (n = 13), suggesting that the related distance *r*_C∼R_ exceeded 102 Å. However, intermolecular FRET between Vα_1C_ and Rβ_2d_ was distinctly revealed in 3 out of 8 cells in a range close to the limits of resolution of the method with an estimate of *r*_V∼R_ = 107±1 Å (Table 1). Taken together, FRET measurements between Rβ and the labeled tails of Vα_1C_C corroborated data on intermolecular FRET obtained with Vα_1C_+α_1C_C+Rβ and demonstrated that (a) calcium channels are in close proximity in the plasma membrane, and (b) both the intra- and intermolecular architecture of Ca_v_1.2 channels depend on the type of Ca_v_β present.

## Discussion

Ca_v_1.2 calcium channels initiate Ca^2+^ signal transduction to many different downstream targets in wide variety of cells. Investigation of factors affecting structural organization of Ca_v_1.2 channels is crucial for better understanding the mechanisms of Ca^2+^ signaling. The tendency of Ca_v_1.2 channels to form clusters in the plasma membrane of different cell types has been poorly investigated. Here we studied effects of three major Ca_v_β subunits on structural organization of recombinant Ca_v_1.2 channels expressed in COS1 cells. Because untransfected COS1 cells do not express endogenous calcium channels, they lack natural intracellular partners (e.g., cardiac RyR2) in proximity of exogenous Ca_v_1.2 channels that might promote their clustering through “junctional” coupling [45]. However, recombinant Ca_v_1.2 channels expressed in COS1 cells establish functional coupling to CREB-dependent transcriptional activation [46], pointing to a physiologically relevant integration of recombinant Ca_v_1.2 into a naturally occurring signaling cascade with Ca^2+^/calmodulin-dependent protein kinase II mediating this activity in native cells [47].

TIRF microscopy revealed clusters of recombinant Ca_v_1.2 channels in the plasma membrane of COS1 cells. The size and the plasma membrane density of the clusters significantly depend on the type of Ca_v_β present. This important observation suggests that the type of Ca_v_β present determines the structure of the Ca_v_1.2 clusters. The average cluster size varies from 360 (β_1b_) to 450 nm^2^ (β_3_). Corroborating reasonable dimensions of these values, a mean size of the Ca_v_1.2 cluster with the major cardiac β_2d_ (430 nm^2^) is within the estimated size range (250–560 nm^2^) of rat ventricular RyR2 clusters [48].

Relative arrangement of α_1C_ and Ca_v_β was estimated with sub-nanometer precision using three-color FRET microscopy in live cells with calcium channels in a stable, inactivated state. Our study revealed that in spite of substantial differences in molecular structure (Figure 6*B*), the intramolecular distance between the α_1C_ subunit tails does not significantly depend on the type of Ca_v_β present. Relative position of Rβ_1b_, Rβ_2d_ and Rβ_3_ did not differ significantly. This is interesting because, unlike β_1b_ and β_3_, β_2d_ has a C-terminal β_2_CED domain, which interacts with the IQ region of the α_1C_ C-tail [44].

Another important observation is that N- and C-termini of α_1C_ and N-termini of Ca_v_β subunits of neighbor channels are in close (<120 Å) proximity to each other, which corroborates with the tendency of Ca_v_1.2 to form clusters. Intermolecular distance between the α_1C_ subunits significantly depends on the type of Ca_v_β and increases from 67 Å in the presence of β_1b_ to 79 Å with β_3_. Measurements of FRET between Rβ and neighbor V/C-α_1C_ supported this general picture and showed a significant effect of the type of Ca_v_β present on the relative position of neighbor channels.

Interestingly, freeze-fracture of the surface membrane revealed that distances between Ca_v_1.2 channels trapped in cardiac junctions with RyR2 is variable and, on average, are larger than those identified by FRET [49]. It is known that the cytoskeleton and RyR2 associate with Ca_v_1.2 plasma membrane clusters in heart cells [50]. Thus, one can not exclude that the distance between Ca_v_1.2 channels in clusters in cardiac junctions is affected by RyR2. However, it is not clear whether clustering affects the ability of Ca_v_1.2 channels to initiate Ca^2+^ signaling and whether every channel is responsive to depolarizing stimuli. In cardiac muscle cells, a single Ca_v_1.2 opening triggers activity of 4–6 RyR2 [51]. The average size of a RyR2 cluster in ventricular myocytes plasma membrane is 250 nm^2^ (∼100 RyR2 molecules) [48] and interaction between Ca_v_1.2 and RyR2 is weaker than that between Ca_v_1.1 and RyR1 in skeletal muscle. Thus, activation of a RyR2 cluster may be mediated by random opening of few Ca_v_1.2 channels in clusters located at a larger distance than that estimated by FRET.

Little is known about molecular determinants underlying physiologically important cluster organization of Ca_v_1.2 channels in neurons [52]. It was shown recently that scaffolding proteins (AKAP79/150 and PDZ) participating in organizing plasma membrane signaling complexes in neurons were not responsible for organizing Ca_v_1.2 channel clusters [53]. The involvement of Ca_v_β in Ca_v_1.2 channel cluster organization, identified in our study, does not contradict the earlier report that the calmodulin-binding IQ region of α_1C_ has a role in Ca_v_1.2 clustering [19]. Because Ca_v_βs interact with IQ [23], [44], it is possible that both act as concerted determinants in Ca_v_1.2 channel clustering.

In conclusion, our study revealed effects of Ca_v_β subunits on the structural organization of Ca_v_1.2 calcium channel in the plasma membrane in the absence of “junctional” interactions. It remains to be seen whether the observed differences in the cluster packing and arrangement of Ca_v_1.2 contribute to the observed differences in calcium signaling among the cell types with preferential expression of a certain type of Ca_v_β [54]–[56].

## Materials and Methods

### Labeling α_1C_ subunit with mVenus and/or mCerulean

To avoid dimerization, only monomeric forms of fluorescent proteins were used. The C-terminus of human Ca_v_1.2 calcium channel α_1C,77_ subunit was amplified by PCR with sense 5′-ctattgaattcgatatcTGCCAGCAGCCTGGTGGAAGCG-3′ and antisense 5′-gtattaccggtggCAGGCTGCTGACGTAGACCCTGC-3′ primers. The PCR fragment was cleaved with ECoRI and AgeI and incorporated into an mCerulean-N1 [57] vector cleaved with the same enzymes, and the 5′-ECoRV/NotI-3′ fragment from the resulting plasmid was then incorporated into α_1C,77_-pCDNA3 cleaved with AleI and NotI, resulting in the mCerulean_C_-α_1C,77_-pCDNA3 plasmid coding for α_1C_C. The 5′-NdeI/KpnI-3′ fragment from mVenus-C1 vector [26] was incorporated into α_1C,77_-pCDNA3 and mCerulean_C_-α_1C,77_-pCDNA3 cleaved with the same enzymes to yield mVenus_N_-α_1c,77_-pCDNA3 and mVenus_N_-mCerulean_C_-α_1c,77_-pCDNA3, respectively, coding for Vα_1C_ and Vα_1C_C.

### Labeling of Ca_v_β subunits with monomeric fluorescent tags

The cDNA of human β_1b_ and β_3_ subunits was cloned from a human heart mRNA (Promega) by a nest RT-PCR strategy. For β_1b_, 5′-GACGGGCAGGGCGCCCACTAC-3′ was used as primer for the reverse transcription, sense 5′-GAGGCTCCTCTCCATGGTCCAG-3′ and antisense 5′-CCACTACATGGCATGTTCCTGC-3′ primers were used for the first round PCR, sense 5′-GCCACCATGGTCCAGAAGACCAG-3′ and antisense 5′-CACTACATGGCATGTTCCTGCTC-3′ primers were used for the second round PCR. For β_3_, primer 5′-CGCCTGTGCCTGCCAGGGTAGGGCAGCAGG-3′ was used for the reverse transcription, sense 5′-GACTCCCCCATGTATGACGAC-3′ and antisense 5′-GGCTGTCAGTAGCTATCCTTG-3′ primers were used for the first round PCR, sense 5′-GCCACCATGTATGACGACTCC-3′ and antisense 5′-TGTCAGTAGCTATCCTTGGGC-3′ primers were used for the second round PCR. The cDNA was cloned into a TA cloning vector pCR 2.1 (Invitrogen) and confirmed by DNA sequencing. The 5′-EcoRV/BamHI-3′ fragment of a β_1b_ TA clone was incorporated into the pTagRFP-C vector (Evrogen, Moscow, Russia), which was cleaved with XhoI, filled in with Klenow and then cleaved with BamHI to generate RFP-β_1b_ (Rβ_1b_). In a similar way the 5′-XhoI/HindIII-3′ fragment of a β_3_-TA clone was incorporated into the pTagRFP-C vector to generate monomeric Rβ_3_. To prepare RFP-β_2d_, β_2d_ was amplified by PCR using mVenus-β_2d_ [44] as template with sense primer 5′-CGGAGATCTATGGTCCAAAGGGACATGTC-3′ and antisense primer 5′-GGGGTCGACTCATTGGGGGATGTAAACATC-3′, and then the PCR product was cleaved with BglII and SalI, and incorporated into the pTagRFP-C vector cleaved with the same enzymes.

### FRET calibration constructs

CTV, C5V, C39V, CVC and VCV were obtained from Drs. Ikeda and Vogel (NIAAA, NIH). The 5′-NheI/BsrGI:(Klenow-filled-in)-3′ fragments of mVenus-C1 and mCerulean-C1 were cloned into pTagRFP-C, which was cleaved with AgeI, filled in with Klenow and then cleaved with NheI, to make V4R and C4R respectively. The 5′-NheI/BamHI:(Klenow-filled-in)-3′ fragment of pTagRFP-C was cloned into mCerulean-N1, which was cleaved with EcoRI, filled in with Klenow and then cleaved with NheI, to make R39C. To prepare R17V and R17C, the 5′-NheI/XhoI:(Klenow-filled-in)-3′ fragment of pTagRFP-C was cloned, respectively, into mVenus-N1 and mCerulean-N1, which were cleaved with BamHI (Klenow filled in) and NheI. CTV was cleaved with BspEI, and the 0.7 Kb fragment was inserted into R17V and R17C to generate RTV and RTC, respectively. mCerulean was amplified by PCR using sense primer 5′-TATATCCGGAGATATCATGGTGAGCAAGGGCGAGGAG-3′ and antisense primer 5′-TATAGAATTCTTTGTACAGCTCGTCCATGCCGA-3′. After cleavage with BspEI and EcoRI, the PCR product of mVenus was inserted into pTagRFP-C to yield R5V; the PCR product of mCerulean was inserted into pTagRFP-C and C4R to yield R5C and CRC, respectively. RFP was amplified by PCR with sense primer 5′-TATAGAATTCGATATCATGGTGTCTAAGGGCGAAGAGCTG-3′ and antisense 5′-ATATGGTACCATTAAGTTTGTGCCCCAGTTTGCTAG-3′, cleaved with EcoRI and KpnI, and incorporated into R5V and R5C cleaved with the same enzymes to yield RVR and RCR, respectively.

### Imaging

Images were recorded with a pixel size of ca. 200 nm using a 14-bit Hamamatsu C9100-12 digital camera (Hamamatsu City, Japan) mounted on a Nikon TE2000 epifluorescent microscope (Tokyo, Japan) equipped with a 60×1.45 numerical aperture (n.a.) oil objective and multiple filter sets (Chroma Technology, Rockingham, VT). Excitation light was delivered by a 175 W xenon lamp. Excitation filter sets were changed by a high-speed filter wheel system (Lambda 10-2, Sutter Instrument, Novato, CA). The Dual-View system (Optical Insights, Santa Fe, NM) was used for the simultaneous acquisition of two fluorescence images (donor and FRET). Images were collected and analyzed using C-Imaging (Compix, Cranberry Township, PA) and MATLAB v.7.0.4 (The Mathworks, Natick, MA).

Two-color FRET was quantified with three filter sets: for the yellow fluorescent protein (YFP) cube, excitation filter 500/20 nm, dichroic beam splitter 515 nm, emission filter 535/30 nm; for the cyan fluorescent protein (CFP) cube, excitation filter 436/20 nm, dichroic beam splitter 505 nm, emission filter 480/40 nm; for the FRET cube (CFP/YFP), excitation filter 436/20 nm, dichroic beam splitter 5051nm, emission filter 540/30 nm. For three-color FRET, the six-filter method described in [39] was used. All FRET images were acquired sequentially. For imaging mCerulean/mVenus pairs, the same filter arrangement as for two-color FRET was used. For the mCerulean/tagRFP combination, the following settings were used: for CFP cube, excitation filter 436/20 nm, dichroic beam splitter 505 nm, emission filter 480/40 nm; for RFP cube, excitation filter 555/28 nm, dichroic beam splitter 565 nm, emission filter 630/50 nm; for the FRET cube, excitation filter 436/20 nm, dichroic beam splitter 565 nm, emission filter 630/50 nm. With the mVenus/tagRFP combination, the following filter arrangement was used: for YFP cube, excitation filter 500/20 nm, dichroic beam splitter 515 nm, emission filter 535/30 nm; for RFP cube, excitation filter 555/28 nm, dichroic beam splitter 565 nm, emission filter 630/50 nm; for the FRET cube, excitation filter 484/15, dichroic beam splitter 565 nm, emission filter 630/50 nm. TIRF images were obtained with TIRF2 Nikon system mounted on Nikon TE2000 microscope and argon-ion laser with 514 nm line and diode laser with 440 nm line, dichroic beam splitter 505 nm, emission filters 470/30 nm and 550/30 nm.

Clusters within TIRF images were identified using 2D continuous wavelet transform similar to [58]. Images were analyzed using a two-dimensional mexican hat wavelet over scales 0.5 through 2 to identify ROI of locally increased signal fluorescence up to 5 µm^2^ in area. Similar approaches have been employed for cluster detection in clinical and cell biology imaging [46], [59], [60]. Corrected FRET intensity was calculated from data acquired using the three filter sets (CFP, YFP, and FRET) as described previously [40] using MATLAB. Briefly, corrected FRET values (*FRET_c_*) were calculated according to
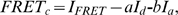
where *a* and *b* are bleedthrough coefficients and *I_FRET_*, *I_d_* and *I_a_* are FRET, donor and acceptor intensities.

Measurement of the *G* factor, which relates the increase in sensitized acceptor emission to the loss of donor fluorescence (quenching), is critical for calculating FRET efficiency (*E*) using the three-filter cube method. *G* factor is a constant for a particular fluorophore pair and imaging setup [42]. This method requires preparation of cDNA constructs encoding donor-acceptor fusion fluorescent proteins differing as widely as possible in FRET efficiency. This was accomplished by varying the length and composition of the linker residues connecting mCerulean and mVenus, mCerulean and tagRFP or mVenus and tagRFP. *G* factor was determined as
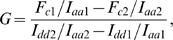
where *I_aa1_*, *I_dd1_* and *F_c1_* are acceptor, donor and corrected FRET intensity of the construct with the shortest linker between donor and acceptor, and *I_aa2_*, *I_dd2_* and *F_c2_* are acceptor, donor and corrected FRET intensity of the construct with the longest linker between donor and acceptor. Using this formula, we found *G* factors of 1.81 for the mCerulean/mVenus pair, 1.30 for the mVenus/tagRFP pair, and 0.38 for the mCerulean/tagRFP pair. These *G* factor values allowed us to calculate FRET efficiency according to [42] as follows:
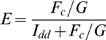



The distance between two fluorophores was calculated in accordance with Förster theory:
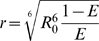



The Förster distances (*R_0_*), the characteristic distance where the FRET efficiency is 50%, was calculated according to [61]:
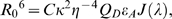
where *Q_D_* is the donor quantum yield, *ε*_A_ is the maximal acceptor extinction coefficient, and *J(λ)* is the spectral overlap integral between the normalized donor fluorescence and the acceptor excitation spectra. All these parameters were calculated based on data obtained from Evrogen for tagRFP and reported for mCerulean and mVenus in [61]. Other parameters included coefficient *C* = 8.786×10^−11^ mol⌖L^−1^⌖cm⌖nm^2^, *κ* ^2^≤2/3 representing the angle between the two fluorophore dipoles assuming random orientation, and η≤1.4, the typical refractive index for biomolecules in aqueous solution [43]. The Förster distance estimated for mCerulean-mVenus was 5.3 nm, while *R*_0_ for mCerulean-tagRFP was 5.1 nm and *R*_0_ for mVenus-tagRFP was 5.8 nm. The *k* factor - the ratio of donor to acceptor (D/A) fluorescence intensity for equimolar concentrations in the absence of FRET, was determined for each construct in accordance with [42]:
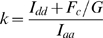



The *k* factor for mCerulean/mVenus was calculated to be 0.41, while mVenus/tagRFP gave *k* = 1.60 and mCerulean/tagRFP gave *k* = 0.27.

D/A ratio for arbitrary concentrations of donor and acceptor was calculated according to [42]:
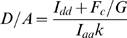



For corrected FRET efficiency measurements, this ratio should be in the range from 0.2 to 5.0 [41]. During analysis, the pixels with D/A ratio outside this range were eliminated from the FRET efficiency calculations.

Validation of *G* and *k* factors is presented in Figure S1 for two- and three-color FRET standards with different FRET efficiencies (linkers) and D/A stoichiometry. In our three-color FRET experiments, the major energy transfer was observed directly between mCerulean and tagRFP and not from cascade transfer through mVenus. If there would be a significant contribution of cascade FRET through mVenus, we would see a decrease in efficiency when we used two-color FRET (mCerulean/tagRFP) compared with three-color FRET, potentially including contributions from mCerulean/mVenus/tagRFP cascade. We did not observe a decrease in efficiency with two-color FRET, as experiments with Rβ_3_ and α_1C_C gave the same efficiency of 0.05 (*r* = 80 nm) as three-color FRET experiments with Rβ_3_, α_1C_C and Vα_1C_. Additional control experiments showed that the third fluorophore did not have a significant effect on mCerulean-mVenus FRET: we did not observe a significant difference between the distance between mCerulean/mVenus fluorophores (73±3, n = 10) measured by two-color FRET with Vα_1C_C and unlabeled β_2d_ and that obtained with three-color FRET using Rβ_2d_ and Vα_1C_C (68±2 nm, n = 13).

For each cell, we calculated FRET efficiency and distances (*r*) between fluorophores in each pixel of ROI. Gaussian fitting of the *r* distribution (20 bin histogram) was done in MATLAB using the fit function:
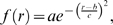
where *b* is the position of the center of the peak (mean) and *c* (dispersion coefficient) reflects the width of the distribution.

## Supporting Information

Figure S1 FRET efficiency and donor/acceptor ratio of FRET standards. Shown are bar graphs summarizing the mean FRET efficiency (A) and the D/A ratio (B) for the indicated FRET calibration constructs. Data are presented as mean±SEM. (A) FRET efficiency values were: C4R (0.110±0.005), R39C (0.081±0.003), CTV (0.023±0.004), V4R (0.433±0.011), RTV (0.191±0.009), C5V (0.474±0.014), C39V (0.266±0.014) and CTV (0.179±0.006). (B) D/A ratios were: C4R (0.99±0.009), R39C (1.00±0.10), RTC (1.00±0.06), CRC (1.96±0.10), RCR (0.48±0.03), V4R (1.00±0.07), RTV (1.00±0.10), RVR (0.53±0.09), C5V (1.00±0.04), C39V (0.95±0.09), CTV (1.00±0.06), CVC (2.00±0.10) and VCV (0.54±0.02). The number of tested cells is shown in the bars. As one can see, increasing the length of the linker between the fluorophores significantly reduced FRET efficiency consistent with an increased distance between donor and acceptor. The measured mean D/A ratio corresponds well to the expected values of 1.0 (1∶1), 2.0 (2∶1) and 0.5 (1∶2). D/A ratios were also determined for the three-color construct CRV. D/A ratio and FRET efficiency were calculated in CRV independently for each pair of fluorophores. D/A ratio (E_FRET_) are: for CV, 1.00±0.07 (0.45±0.02); for VR, 1.10±0.10 (0.41±0.02), and for CR, 0.99±0.07 (0.20±0.01), n = 11. Thus, the D/A ratio of three-color standards well corresponds to the expected 1∶1 ratio.
(0.29 MB EPS)
